# Biomechanical and Physiological Evaluation of Respiratory Protective Equipment Application

**DOI:** 10.2147/MDER.S370142

**Published:** 2022-07-26

**Authors:** Silvia Caggiari, Dan L Bader, Finn Foxell, Nicholas Pipe, Seana Couch, Abbie Turner, Peter R Worsley

**Affiliations:** 1 Clinical Academic Facility, School of Health Sciences, University of Southampton, Southampton, SO17 1BJ, UK

**Keywords:** respiratory protective equipment, skin health, goodness of fit, interface pressure, physiological response

## Abstract

**Purpose:**

Respiratory protective equipment is widely used in healthcare settings to protect clinicians whilst treating patients with COVID-19. However, their generic designs do not accommodate the variability in face shape across genders and ethnicities. Accordingly, they are regularly overtightened to compensate for a poor fit. The present study aims at investigating the biomechanical and thermal loads during respirator application and the associated changes in local skin physiology at the skin–device interface.

**Materials and Methods:**

Sixteen healthy volunteers were recruited and reflected a range of gender, ethnicities and facial anthropometrics. Four single-use respirators were evaluated representing different geometries, size and material interfaces. Participants were asked to wear each respirator in a random order while a series of measurements were recorded, including interface pressure, temperature and relative humidity. Measures of transepidermal water loss and skin hydration were assessed pre- and post-respirator application, and after 20 minutes of recovery. Statistical analysis assessed differences between respirator designs and associations between demographics, interface conditions and parameters of skin health.

**Results:**

Results showed a statistically significant negative correlation (p < 0.05) between the alar width and interface pressures at the nasal bridge, for three of the respirator designs. The nasal bridge site also corresponded to the highest pressures for all respirator designs. Temperature and humidity significantly increased (p < 0.05) during each respirator application. Significant increases in transepidermal water loss values (p < 0.05) were observed after the application of the respirators in females, which were most apparent at the nasal bridge.

**Conclusion:**

The results revealed that specific facial features affected the distribution of interface pressures and depending on the respirator design and material, changes in skin barrier function were evident. The development of respirator designs that accommodate a diverse range of face shapes and protect the end users from skin damage are required to support the long-term use of these devices.

## Introduction

Since the outbreak of COVID-19, healthcare workers have been required to use respiratory protective equipment (RPE) for prolonged periods, for example a 12-hour shift which is often repeated over consecutive days. Altough the use of respirators minimises the risk of transmission of COVID-19^1^ adverse skin reactions have been reported associated with their prolonged use, and there are reports of fitting issues which are shown to affect 42–97% of clinical personnel. A recent review reported that the skin damage detected in medical staff were predominantly located on the nasal bridge and cheeks, including pressure-induced damage, moisture-associated skin dermatitis and skin tears, all associated with RPE devices.^2^ In addition to the direct injury, skin irritation, itchiness and dry skin were commonly reported. Indeed, a cumulative effect of repetitive RPE usage was observed to increase the response rate of reactions, which could be associated with a decrease in skin tolerance over time. Unfortunately, these events do not represent a new occurrence. Indeed, skin damage from medical devices have been widely documented among patients and healthcare providers.^3^ In addition, there have been reports of ill-fitting RPE devices for particular sub-groups of the population who showed higher rates of adverse skin reactions, prior to the outbreak of the pandemic.^4^

Correctly fitting RPE is critical for filtering efficiency that N95 or FFP3 respirators aim to provide. Indeed, it is important for efficient device function that there is no loss of seal at the face–respirator interface. However, creating an effective seal between the device and the face is challenging due to the variability in face shape and the limited number of geometries, sizes, and materials in the current commercial respirator market. For example, the standards (EN-149) by which many respirators are manufactured relies on a panel of anthropometric features derived from a white male cohort.^5^ Thus, the current design template provides limited diversity in terms of size and geometry, and it is often unable to accommodate specific facial shapes and asymmetry. As a result, several studies have reported that females and those from Black and Asian ethnic minority groups have a lower success rate in respirator fitting.^6^ Thus, there is a critical need to improve the design criteria of RPE to provide a range of respirators to accommodate the user population. This could provide a means to address the root cause of ill-fitting devices.

Individuals with poorly fitting RPE devices regularly over-tighten the straps, resulting in high non-uniform pressures, particularly at bony locations of the face, such as the bridge of the nose.^7^ This highlights the importance of an effective fitting process prior to use.

Recent studies have examined the relationship between facial dimensions and goodness-of-fit (GoF) in different sub-populations, identifying specific facial measures as predictors of fitting outcomes.^8^,^9^ However, this precludes generalising the findings to a wider population reflective of the healthcare workforce, which is characterised by gender and ethnicity diversity. In addition, there is limited knowledge of the association between GoF and markers of skin health, as result of RPE application. There are several measurement approaches to characterise the boundary between a device and the skin interface, and to monitor the skin response following application.^10^ These parameters include interface pressures, microclimate and skin physiology at the face–respirator interface. The combination of these measurements has been used to monitor tissue viability at the medical device interface, for example during the application of non-invasive ventilation masks (NIV).^11^ Indeed, it has been reported that prolonged pressure and excess moisture alone, or in combination, can compromise the integrity of the skin barrier.^12^

Recent studies investigated the effect on skin physiology and microclimate of a single-use respirator device of the type KN95 or KF94.^13–15^ Results showed changes in the skin barrier function and microclimate at specific facial locations such as the cheek, even after relatively short periods. An accumulation effect was also observed, with skin physiology parameters and temperature increasing over time, and returning to baseline levels after prolonged periods of non-use (>12 hours).^13^,^14^ Limitations of these studies include the fact that the nasal bridge was not included as investigation site, despite being a frequently reported site of RPE-related facial injuries.^11^ By contrast, Peko et al^15^ investigated the contact force at the respirator interface at both nasal bridge and cheeks, with the latter subjected to a higher force. However, investigating a single respirator design precludes generalising findings to the variety of RPE devices that are used by the healthcare workforce. In addition, the aforementioned studies were conducted on single ethnic populations, such as Korean or White Caucasian, limiting the comparison between ethnic groups and not reflecting the diversity in ethnic background of the healthcare workforce population.^6^,^16^

This motivated the present study, which aimed to evaluate the biomechanical, thermal and physiology responses at the face–respirator interface following the application of four different RPE devices.

## Materials and Methods

A randomized crossover design study with a cohort of health volunteers was used in the present study. The study follows the guidelines outlined in the Declaration of Helsinki.

### Participants

A convenience sample of participants reflecting different genders and ethnic backgrounds was recruited from the local university population. Participant recruitment was conducted by means of poster advertisements and word of mouth. Exclusion criteria involved past or present skin conditions, including skin diseases or allergies. Written informed consent was obtained from each participant prior to testing. Ethics approval was granted by the University of Southampton Ethics Committee (FOHS-ERGO-61418).

### Test Equipment

Interface pressures were monitored using a commercial system (Mk III; Talley Medical, Romsey, UK), which incorporated individual 18 mm diameter cells with a reported mean error of 12 ± 1% and repeatability of ±0.53 mmHg.^17^ Microclimate at the device–skin interface was recorded using combined sensors (SHT75; Sensiron AG, Switzerland), sampling relative humidity and temperature at a frequency of 1 Hz. The sensors have a reported accuracy of ±0.5% RH and ±0.8℃, respectively.

Biophysical skin properties before and after respirator application were measured with an array of tools. This included the skin barrier function assessed using an open-chamber probe, measuring transepidermal water loss (TEWL) at a sampling frequency of 1 Hz (TM 300w, Courage & Khazaka Electronic GmbH, Germany).^18^ The moisture content of the stratum corneum (skin hydration) was also recorded using a probe assessing the dielectric constant of the skin at a depth of 10–20 µm (Corneometer, CM 825, Courage & Khazaka Electronic GmbH, Germany). Both probes were applied to the skin surface, ensuring gentle contact whilst recording.

A white light non-contact handheld scanner (GoScanner, Creaform) was used to capture 3D facial scans of each participant, prior to respirator application. The scanner has been shown to have a surface height error magnitude of up to 0.33 mm, across 95% of the target surface.^19^

### Respirators

Four single-use commercial FFP3 respirators, representing the most frequently supplied at regional hospital sites, were carefully chosen to reflect different geometries, sizes and material interfaces. Two respirators were characterised by a similar unvalved 3-panel foldable geometry, representing a small (R1) and a large (R3) size. By contrast, the other two respirators were characterised by an unvalved (R2) and a valved (R4) rigid shell geometry, respectively. The latter incorporated self-tightening straps (R4). For all respirators, participants were instructed to pull the bottom and top straps over the head, place the former around the neck, below the ears, and the latter on top of the head, according to manufacturer’s guidelines for application. For the respirator with self-tightening straps, participants were instructed to tighten them until they felt a balance between comfort and seal. Where relevant, participants were asked to mould the nosepiece to the shape of their nose and thereafter to cover the respirator completely with hands and exhale sharply. If air escaped, they were asked to readjust the respirator, tightening the straps where applicable.

### Test Protocol

A test protocol involving two 90-minute sessions was performed at the Biomechanics Testing Laboratory at the Clinical Academic Facility of the Southampton General Hospital, where ambient temperature was maintained at 20°C±2°C and relative humidity ranged between 42% and 57%. In all testing sessions, participants were required to attend with clean washed skin and have, where appropriate, shaved at least 48 hours before the session. Demographic data including age, height, weight, gender and ethnic background were collected at the beginning of the first visit. In addition, a facial scan was taken for each participant (pre-respirator application).

**Figure 1:**
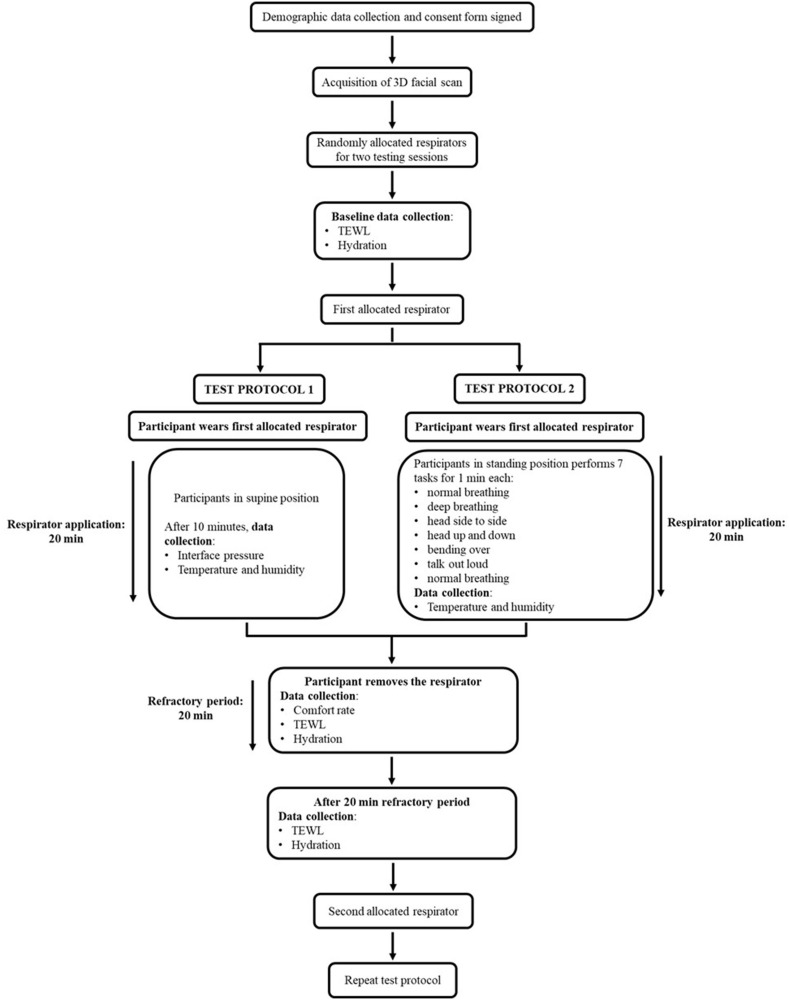
Schematic depicting the two testing protocols and describing the test procedures involved.

The two testing sessions were conducted at least 48 hours apart with a random allocation of two respirator models at each testing session. During the corresponding testing sessions, each participant was asked to wear two respirator models, as instructed per manufacturer’s guidelines, for a period of 20 minutes in accordance with the test protocol depicted in Figure 1. The test protocol was modified during the respirator application phase for half of the cohort, to compare static postures (protocol 1) and activities during respirator donning (protocol 2).

A series of baseline measurements of TEWL and skin hydration were performed at two investigation sites, namely the nasal bridge and the right cheek bone, both of which are contacted by RPE devices and have been associated with respirator-related skin damage.^20^ In addition, the right pre-orbital area was measured to represent an unloaded control site, selected to be a minimum of 30 mm distal to the site of respirator loading. Participants then donned one of the respirator models and were asked to adopt a supine posture while breathing normally. After 10 minutes, three interface pressures averaged over a 2-minute period were recorded at each investigation site, with a pressure sensing cell placed at the face–respirator interface. A combined microclimate sensor was then placed at the respirator–skin interface, where temperature and humidity were recorded over a 1-minute period. Following the 20-minute period of application, each participant was asked to rate their level of comfort on a 10-point visual analogue scale, with 0 representing no discomfort and 10 extreme discomfort.^21^ This was subsequently followed by a 20-minute refractory period with no respirator, after which post-recovery values of TEWL and hydration were recorded.

TEWL was recorded for a period of approximately 1 minute, sufficient to attain equilibrium, with the mean of the last 10 values used as the output parameter. By contrast, assessment of hydration involved the mean of 5 consecutive measures. All measurements were then repeated for the second randomly allocated respirator model.

During test protocol 2, the participants were asked to perform seven different tasks whilst wearing the respirator, which are used during the fit2fit quantitative fit test.^22^ These included normal breathing, deep breathing, head side to side, head up and down, bending over, talk out loud, and normal breathing, each for a 1-minute period. These exercises emulate a full range of facial movements. During each movement, the combined microclimate sensor recorded interface temperature and humidity. It was not possible to measure interface pressures reliably during these dynamic movements.

### Data Processing and Analysis

3D facial scans of each participant were retrieved through the proprietary software (VXelements, Createform), subjected to a cleansing process to eliminate unnecessary elements, eg, background, and converted to binary.stl files. These were then imported into a modified version of the python module AmpScan^23^ to estimate five facial anthropometrics, namely, facial length, 1/3 facial length, alar and bio-ocular width, and dorsal nasal length. For each measurement, two appropriate points were selected by the same investigator, and their distance was calculated on the facial scan.

Statistical analysis was performed using IBM SPSS statistics V22 (IBM Corp, Armonk, NY, USA). Data from each of the test parameters were examined for normality using Shapiro–Wilk tests. Parametric descriptors (mean ± SD) were found to be appropriate for the analysis of TEWL and hydration, interface pressure, microclimate and facial anthropometrics. Non-parametric descriptors were used for subjective comfort. Pearson correlation was used to evaluate associations between pressure values and facial dimensions. Since interface pressures were collected only during static postures while respirators were applied, data are presented only for protocol 1.

A paired *t*-test was performed to assess the difference between test protocols and the effects of respirator types on the outcome measures. This included the differences in macroclimate at respirator interface compared to ambient conditions, for all respirators. It was also performed to assess variations in TEWL and hydration, pre- and post-respirators application, and following the refractory period.

As opposed to pressure, microclimate, TEWL and hydration data are presented for both protocols. The analyses involving differences in TEWL and hydration with respect to gender and ethnicity will be presented for the entire cohort of sixteen participants. For all outcomes, the statistical significance level was set at 5% level (*p* ≤ 0.05).

## Results

### Participants

Sixteen healthy participants (eight males and eight females) were included in the study, equally divided between test protocols 1 and 2 (Table 1). The former included five males and three females, aged between 29 and 40 years (mean = 36 years) with a mean height and weight of 1.70 ± 0.1 m and 71.0 ± 24.1 kg, respectively. The corresponding BMIs ranged between 17 and 35 kg/m^2^. By contrast, protocol 2 included three males and five females, aged between 20 and 34 years (mean = 22 years), with an average height, weight, and BMI approximately equivalent to participants in protocol 1 (Table 1). Both protocols included participants with a range of ethnicities.

**Table 1:** Summary of Demographic Data of Participants Involved in Each Protocol

Protocols	Participants	Gender	Ethnicity	Age (y)	Height (m)	Weight (kg)	BMI (kg/m^2^)
Protocol 1	1	Male	Black African	31	1.74	76.6	25.3
	2	Male	Black African	40	1.68	60.9	21.6
	3	Male	Asian	38	1.82	79.9	24.1
	4	Male	Asian	36	1.77	81.1	25.9
	5	Male	White Caucasian	37	1.85	119.0	34.8
	6	Female	White Caucasian	35	1.55	49.0	20.4
	7	Female	Asian	39	1.56	41.5	17.1
	8	Female	Asian	29	1.59	60.5	23.9
***Mean values***				35.6 (±3.9)	1.70 (±0.1)	71.1 (±24.1)	24.1 (±5.2)
Protocol 2	1	Female	White Caucasian	21	1.62	68.0	25.9
	2	Female	White Caucasian	20	1.76	76.2	24.6
	3	Female	White Caucasian	21	1.72	68.8	23.3
	4	Male	Mixed	21	1.76	89.1	28.8
	5	Male	White Caucasian	20	1.82	78.7	23.8
	6	Male	White Caucasian	20	1.72	72.5	24.5
	7	Female	Mixed	20	1.62	72.2	27.5
	8	Female	Black African	34	1.66	66.8	24.2
***Mean values***				22.1 (±4.8)	1.71 (±0.1)	74.0 (±7.3)	25.3 (±1.9)

### Interface Pressure

Results revealed that the pressure values at the bridge of the nose were higher than the corresponding values at the cheek for all four respirators, with significant mean differences ranging between 46 and 77 mmHg (p < 0.05). A statistically significant (p < 0.05) difference between R1 and R3 was found in the pressure values at the nasal bridge. These respirators correspond to the unvalved 3-panel foldable design.

**Figure 2:**
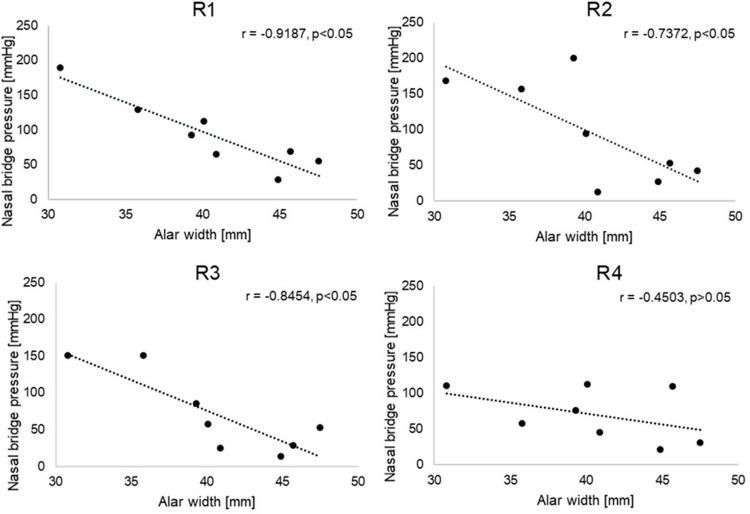
Relationship between interface pressures recorded at the nasal bridge and alar width, for all the respirators. R1 and R3 correspond to the unvalved 3-panel foldable design, R2 and R4 are characterised by a rigid shell geometry.

When the interface pressures at the nasal bridge were compared against the alar width of participants, the resulting associations yielded a statistically significant negative correlation (p < 0.05), for three of the respirator designs (Figure 2). The wider noses resulted in the lower pressure values as indicated in Figure 2.

### Temperature and Humidity

Results revealed that during respirator application the skin interface had a mean temperature of between 32°C and 35°C, and to a relative humidity of between 68% and 77%, for both protocols (Table 2). When compared to the corresponding ambient conditions, the differences were statistically significant (p < 0.05). No differences were found between respirator designs or test protocols. It is of note that the ambient humidity in protocol 1 was higher than in protocol 2, but the resulting relative humidity at the respirator interface was approximately equivalent.

**Table 2:** Summary (Mean (±SD)) of Microclimate for Both Ambient and Respirator in situ Conditions, for All Respirators and Testing Protocols

		Temperature [°C]		Humidity [%]	
	Respirators	Ambient	Respirator in Situ	Ambient	Respirator in Situ
Protocol 1	*R1*	22.4 (±1.9)	33.3 (±1.5)*	56.9 (±6.4)	73.4 (±7.1)*
	*R2*	22.6 (±1.9)	33.8 (±1.1)*	57.1 (±7.7)	72.8 (±4.5)*
	*R3*	22.4 (±1.9)	34.2 (±1.2)*	54.4 (±5.3)	68.2 (±3.3)*
	*R4*	22.9 (±1.7)	33.0 (±1.8)*	54.5 (±6.3)	72.2 (4.5)*
Protocol 2	*R1*	24.0 (±2.0)	32.7 (±1.0)*	42.2 (±7.2)	76.6 (±7.1)*
	*R2*	24.9 (±1.8)	33.9 (±0.8)*	46.5 (±5.0)	75.6 (±4.4)*
	*R3*	24.1 (±1.5)	32.3 (±1.2)*	45.4 (±5.1)	72.8 (±3.5)*
	*R4*	23.9 (±2.9)	34.6 (±0.6)*	48.6 (±7.1)	72.7 (±6.5)*

**Note**: *Indicates a statistically significant differences compared with ambient measures (p<0.05).

### TEWL and Hydration

Results showed no statistically significant differences between the protocols for TEWL and hydration, corresponding to all the respirators. Table 3 summarises the TEWL absolute values collected at both test sites pre- and post-respirator application and following the refractory period. TEWL values were higher at the nasal bridge compared with the baseline, which were statistically significant (p < 0.05) for two of the respirator models during protocol 1 and three models in protocol 2. Differences in the magnitude were relatively small, particularly in protocol 1. By contrast, in protocol 2, R2 and R4, which are both characterised by a rigid shell, yielded the highest increase, of 9.3 and 4.7 g/h/m^2^, respectively. Following the refractory period, TEWL at the nasal bridge reduced to values similar to baseline, indicating a partial or full recovery of the skin barrier function. There was also an increase in TEWL values at the cheek following respirator application, with differences varying between respirator designs and test protocols (Table 3). Altough following the refractory period the TEWL reduced to values close to baseline for protocol 2, they remained significantly higher (p < 0.05) for R1 and R4, in protocol 1. TEWL values recorded at the control site following respirator application and refractory period did not show significant differences with respect to the baseline values (data not shown).

**Table 3:** Summary (Mean ±(SD)) of TEWL Values, in g/h/m2, Collected at the Nasal Bridge and Right Cheek, Pre- and Post-Respirator Application, and Post Refractory Period

		TEWL [g/h/m^2^]					
		Nasal Bridge			Right Cheek		
	Respirators	Baseline (B)	Post Respirator (PR)	Post Refractory Period (PRP)	Baseline (B)	Post Respirator (PR)	Post Refractory Period (PRP)
Protocol 1	*R1*	19.2 (±3.2)	18.7 (±3.1)	19.3 (±4.6)	19.0 (±7.2)	21.0 (±7.2)	22.1 (±7.8)*
	*R2*	19.8 (±2.7)	23.7 (±4.2)*	19.6 (±4.6)	19.2 (±6.8)	20.1 (±7.3)	22.8 (±9.2)
	*R3*	19.6 (±5.1)	21.4 (±4.3)	18.3 (±5.5)	20.6 (±8.2)	23.7 (±7.6)*	22.1 (±8.3)
	*R4*	19.7 (±4.8)	21.9 (±4.8)*	19.0 (±4.4)	20.7 (±7.8)	23.9 (±8.3)*	23.9 (±8.4)*
Protocol 2	*R1*	19.0 (±3.5)	22.0 (±3.9)*	18.5 (±3.3)	22.1 (±3.6)	22.3 (±2.6)	23.6 (±3.6)
	*R2*	24.6 (±7.8)	33.9 (±9.8)*	22.3 (±6.0)	21.9 (±3.1)	24.5 (±2.2)*	21.3 (±9.0)
	*R3*	20.4 (±6.8)	21.1 (±3.7)	20.2 (±4.4)	22.4 (±2.8)	23.4 (±2.5)	22.2 (±3.1)
	*R4*	20.8 (±5.2)	25.5 (±5.2)*	20.3 (±8.7)	23.8 (±4.1)	23.1 (±2.7)	23.4 (±3.3)

**Note**: *Indicates a statistically significant differences compared with baseline (p<0.05).

Results also showed a significant increase in skin hydration at both nasal bridge and cheek sites, following the application of R2 and R4 in protocol 1 (data not shown). A statistically significant increase at the cheek was also recorded for R3. By contrast, in protocol 2 only R2 demonstrated a significant higher value at the nasal bridge when compared to the baseline (p < 0.05). Analysis of post-refractory period showed statistically significant higher values at the cheek for all respirators used in protocol 1 (data not shown). Hydration values at the control site showed a statistically significant increase, in three of the respirators during protocol 1 (p < 0.05). By contrast, protocol 2 did not show any significant changes in control site (p > 0.05).

**Figure 3:**
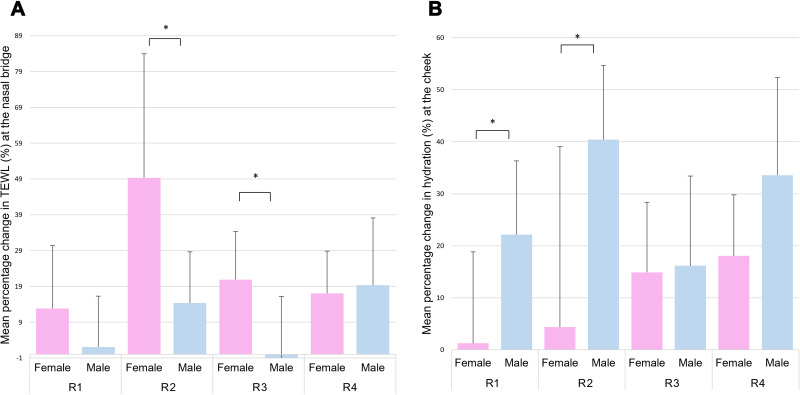
(**A**) TEWL percentage increments at the nasal bridge for each of respirators in both protocols with respect to gender. (**B**) Skin hydration percentage increments at the cheek for all respirators with respect to gender. R1 and R3 correspond to the unvalved 3-panel foldable design, R2 and R4 are characterised by a rigid shell geometry. *Indicates a statistically significant difference (p<0.05).

Closer examination of the data collated from both protocols revealed gender-specific changes in TEWL and hydration (Figure 3A and B). Results showed that the percentage changes in TEWL were statistically higher for the females for R2 and R3, with differences in excess of 20%. By contrast, there was a minimal difference between genders following the application of R4 (p > 0.05). The reverse trend was revealed with skin hydration at the cheek, with higher changes associated to male participants with differences ranging from 1% to 36% (Figure 3B). Statistical analyses revealed significant differences corresponding to R1 and R2 (p < 0.05).

### Perceived Comfort

R2 and R4, which are both rigid shell models, produced the lowest comfort score across the participants with median values of 6.0 and 5.0, respectively. Most of the individuals expressed a score equal to or greater than 5 for both respirators. By contrast, only a few individuals reported these values for R1 and R3, which showed a comfort score equal to 4.0. There was no statistically significant difference in subjective comfort score between the two protocols, for three respirators (p > 0.05). Closer examination of the data revealed a statistically significant lower comfort in the female group for R2 (p < 0.05).

## Discussion

The present study used an established array of parameters to investigate the biomechanical and thermal load (pressure and microclimate) associated with respirator application. The corresponding changes in the skin physiology were monitored pre- and post-respirator application to evaluate changes in skin barrier function (TEWL) and hydration. Four respirators reflecting a range of different design principles were tested using two test protocols (Figure 1). The results revealed that specific facial features affected the distribution of interface pressures and depending on the respirator design and material, changes in skin barrier function were evident, which were gender dependent. These changes were largely recovered following a period of respirator removal.

Results revealed that the nasal bridge is the site exposed to the highest mean interface pressures (>70 mmHg), for all respirators. This corresponds to the typical location of RPE-related facial injuries reported in the literature.^16^,^24^ Indeed, this location has minimal soft tissue coverage, creating a point of limited soft tissue conformity at the device interface. It also represents a site of limited tolerance to mechanical loads, where skin is deformed against bone and cartilage sub-dermal structures. Direct comparison with previous studies is limited by the nature of the pressure sensing device. In a recent study, Peko et al^15^ investigated the contact force at both the nasal bridge and cheeks. However, as opposed to our findings, they reported that cheeks were subjected to a higher force.

Our results also show a high degree of variability in the pressure values, particularly evident at the nasal bridge, for all respirators. This could be explained by the inability of the respirators to accommodate the considerable inter-individual differences in face and nose shapes. Indeed, a recent review reported an association between gender- and ethnicity-based facial anthropometric differences and RPE performance,^6^ with specific facial parameters able to predict fitting outcomes in sub-populations.^8^,^9^

To the authors’ knowledge, there are no previous studies that explored the relationship between facial measures and interface pressure, resulting from respirator application. Indeed, our results showed an association between pressures at the nasal bridge and alar width (p < 0.05), with the wider noses resulting in lower interface pressures, as depicted in Figure 2. These geometric findings correspond to the contact mechanics between the deformable mask and the bony prominence of the nose, where a narrow alar shape creates a focal point for load over the nasal bridge. By contrast, a wider, flatter nose will create contact on the sides of the nose and cheeks, offloading the nasal bridge where masks cannot conform to the geometry of the face. The different design of respirators can also contribute to the response to pressure. Indeed, a proportion of respirator models include a flexible aluminium or steel nose clip, which can affect the pressure distribution over the nasal bridge. This is reflected in our results, which showed a statistically significant (p < 0.05) difference in the pressure values at this site, between R1 and R3, which correspond to the unvalved 3-panel foldable design with steel nose clip but are of different sizes. Closer examination of the data showed that the wider noses were associated with non-Caucasian male participants. Despite increased awareness of ethnicity- and gender-based correlations between facial geometries and RPE fit, literature reveals a limited number of studies depicting a correlation between nose width and RPE performance.

Microclimate conditions at the interface between the skin and respirator revealed that temperature and humidity values were 8°C and 15% higher than the ambient conditions, resulting in values in excess of 32°C and 68%, respectively. These findings reflect the results from a recent study,^15^ where significant changes in microclimate were found after 2-hour period of application of a single-use respirator. Our findings also shows that, even during a static posture, all respirator models yielded an increase in temperature and humidity to values that have a detrimental effect on the skin.^25^ Indeed, it is acknowledged that high temperature and excessive moisture lower the skin tolerance to mechanical load, increasing the risk of tissue damage,^26^ specifically at anatomical sites such as the bridge of the nose. In addition, local relative motion between the respirator and skin as a result of specific activates, such as speaking, causes rubbing of the RPE against the skin, which coupled with an accumulation of moisture will create an increase in frictional forces at the skin interface.^27^ Excessive accumulation of moisture might also impact on the fitting of respirators, compromising their efficacy.^28^ By implication, interface materials influence the skin conditions, contributing to lowering its tolerance.

Literature reveals a limited number of studies investigating changes in the skin barrier function following prolonged application of RPE devices.^13^,^14^,^29^ Previous results reported an increase in both TEWL and skin hydration after RPE application, of a similar magnitude to the present study (Table 3), albeit at varying periods of application ranging from 20 minutes to 8 hours. However, these studies did not investigate the skin at the nasal bridge, which has been shown to represent a vulnerable site during respirator application.^16^ In addition, limited analysis exists in evaluating the effects of gender and ethnicity. Our study revealed greater changes in the skin barrier function at the nasal bridge for females, for R2 and R3 (Figure 3A). The former model (R2), which corresponded to a rigid shell design, particularly affected the changes in TEWL, where values were significantly higher than the other three respirators across the female cohort. By contrast, for R1 and R2, males had higher hydration values with no significant differences between respirator models (Figure 3B). Based on the findings of this study, there is a need to evaluate the performance of RPE devices with consideration of gender and ethnic background of users. Indeed, our findings demonstrated that both characteristics can influence the biomechanical and physiological responses of the skin at the respirator interface. The design principles also influenced these responses, with rigid shell designs yielding the highest TEWL increase. In addition, facial measurements, as for example the alarwidth, appear to have an association with the distribution of load, indicative of the quality of the fit. Indeed, a recent review by the authors demonstrated a variance in anthropometric measurements, with female and specific ethnic groups showing smaller facial dimensions and a corresponding lower success rate in fit testing.^6^ In addition, the current fit panels are not able to accommodate those individuals with extremes of BMI.^30^ Indeed, inthose with low BMI bony prominences can create a less conforming surface and gapping, which limits the creation of an airtight seal.

Therefore, we strongly suggest that a collaboration with manufacturers is required to identify new designs and create standards which accommodate face shapes of different genders and ethnicities typically encountered in healthcare settings.

## Limitations

The present study included some methodological limitations related to a relatively short respirator application time of 20 minutes, compared to the prolonged period (>12 hours) healthcare workers are required to wear RPE devices during a working shift. Participants were relatively young healthy volunteers, particularly for protocol 2 (Table 1), which limits the generalisation of the findings. In addition, neither of the protocols adopted activities which truly reflected the physical tasks healthcare workers are exposed to during a working shift. Further limitation of the study involved the inability to reliably measure the interface pressure during the dynamic movements. Further research is needed to investigate the biomechanical, thermal and physiological responses from the application of a wider range of respirator models on an extended larger cohort, reflective of the healthcare worker population.

## Conclusion

The present study has investigated biomechanical, thermal and physiological responses to the application of RPE devices of different designs and materials in a cohort of healthy participants. The study revealed that specific facial features, such as the alar width, affected the distribution of interface pressures, particularly at the nasal bridge. In addition, gender-based changes in skin barrier function were evident. The development of respirator designs that accommodate a diverse range of face shapes is required to support the long-term use of these devices among healthcare workers, who are required to wear the respirators for prolonged time.
